# Four-modal device comprising optical coherence tomography, photoacoustic tomography, ultrasound, and Raman spectroscopy developed for in vivo skin lesion assessment

**DOI:** 10.1364/BOE.559842

**Published:** 2025-04-04

**Authors:** Anatoly Fedorov Kukk, Di Wu, Rüdiger Panzer, Steffen Emmert, Bernhard Roth

**Affiliations:** 1 Hannover Centre for Optical Technologies, Leibniz University Hannover, Nienburger Straße 17, 30167 Hannover, Germany; 2 Clinic and Policlinic for Dermatology and Venereology, University Medical Center Rostock, Strempelstraße 13, 18057 Rostock, Germany; 3 Cluster of Excellence PhoenixD (Photonics, Optics and Engineering - Innovation Across Disciplines), Welfengarten 1a, 30167 Hannover, Germany

## Abstract

The accurate determination of the depth of the infiltration and the pathophysiology is critical to the treatment and excision of skin cancer, particularly melanoma. The current gold standard in skin cancer diagnostics comprises skin biopsy followed by histological examination, which is invasive, time-consuming, and limited in measuring at the deepest level. Non-invasive imaging techniques like dermoscopy, confocal microscopy, and multiphoton microscopy also face limitations in accurately capturing contrast and depth information across lesion locations or distinguishing between malignant and benign tissue. Thus, there is a need for non-invasive techniques capable of obtaining 3D images of skin lesions with sufficiently high spatial resolution, which at the same time assess pathophysiology. To address this problem, we have developed a device that combines optical coherence tomography (OCT), photoacoustic tomography, ultrasound, and Raman spectroscopy into a single scanning unit, enabling the *in vivo* acquisition of co-localized 3D images and Raman fingerprint spectra of skin lesions with all four modalities under 5 minutes for the whole procedure. We performed measurements on 52 suspicious human skin lesions that were immediately excised following the measurements. The resulting lesion depth exhibited a strong correlation with histological thickness between 0.1-5 mm, achieving a coefficient of determination (R^2^) of 0.97, which is higher than those from the individual modalities and from previous studies. In addition, the Raman modality offers the possibility to distinguish between malignant and benign lesions. Our results indicate that the developed multimodal approach for 3D imaging and lesion assessment can offer all the required information for non-invasive skin cancer diagnostics. Further clinical measurements are required to determine diagnostic accuracy, which will be performed in the next step in the case of melanoma skin cancer.

## 1. Introduction

Skin cancer is the most common type of cancer worldwide, with an estimated 1.5 million cases in 2020, including 325,000 new cases of melanoma, which resulted in 57,000 deaths [1]. Although skin cancer is typically regarded as less dangerous than other forms of cancer, such as lung or breast cancer, some variants of it can be equally lethal. Melanoma, in particular, has the potential to metastasize to nearby lymph nodes, the bloodstream, and surrounding tissue, resulting in fatal outcomes if not treated promptly. Early-stage melanoma has a 10-year survival rate of 95%, whereas metastatic melanoma in the late stages have a survival rate of less than 10% [2–5]. Therefore, early detection and treatment are crucial for successful therapy. The current diagnostic method of biopsy and histopathological analysis is invasive, costly, and time-consuming. Additionally, histopathological analysis only evaluates 2D depth images, which may undergo mechanical deformation during excision and processing, potentially leading to inaccurate measurements of lesion depth [6].

Non-invasive imaging techniques, such as confocal microscopy, reflectance confocal microscopy, multiphoton microscopy (MPM), optical coherence tomography (OCT), reflection confocal laser microscopy (RCM), and line-field confocal OCT (LC-OCT) have been explored for skin cancer diagnosis so far. However, these techniques are limited by their penetration depth in highly pigmented skin, typically less than 500 µm [7–15], some of them even to less than 200 µm [16], or extremely long acquisition times, especially for microscopy measurements [17]. In addition, while in the case of the more recent LC-OCT technology, three-dimensional imaging in real time is possible and an artificial intelligence (AI) assistance system integrated, differential diagnosis of nevi and melanomas is not achieved so far. Especially, reliable determination of tumor thickness in melanomas in the range around 1 mm is not reliably possible with OCT and high-frequency ultrasound to date, see e.g. [11]. For deeper lesions, ultrasound (US) imaging has shown good agreement with histological results, particularly with frequencies of 15 MHz or higher [18–22]. Raman spectroscopy (RS), on the other side, has demonstrated an additional advantage compared to other techniques, namely the ability to distinguish between malignant and benign skin lesions by analyzing biochemical composition [23–28]. Also, convolutional neural networks, among others, were applied to improve the classification of tissue based on the Raman spectra obtained from skin lesions (or other types of tissue), in part also in combination with modalities such as OCT [29–32]. However, it was found that so far these combinations do not achieve a sufficient classification accuracy and also that these cannot easily examine thicker skin lesions in the range of 1 mm and above.

Consequently, despite the above advancements, no single modality or combination of modalities can non-invasively diagnose skin cancer and measure lesion thickness across all ranges (50 µm to 5 mm). As consequence, none of the approaches reported to date could be established in clinical use as support or alternative for the current diagnostic gold standard. A multimodal approach combining OCT, photoacoustic tomography (PAT), US, and RS could overcome individual limitations and provide comprehensive diagnostic information to support dermatologists and improve diagnostics. This work presents a novel device that integrates these four modalities into a single unit with a water tank adapter and acoustical mirror, allowing for co-localized measurements without switching of the measurement head. This design can facilitate accurate, non-invasive imaging of skin lesions, potentially improving early diagnosis and treatment outcomes. The integrated multimodal device was tested both on *ex vivo* melanoma tissue and *in vivo* on human nevi including melanoma to evaluate its functionality. In *ex vivo* measurements, the system's imaging capabilities are compared to the histological results, while *in vivo* measurements on human nevi indicate the system’s applicability in clinical scenarios. The setup offers the potential for further miniaturization and integration so that it can be an interesting system for future skin diagnostics.

## 2. Experimental setup

### 2.1. Combined multimodal system

**Fig. 1.**
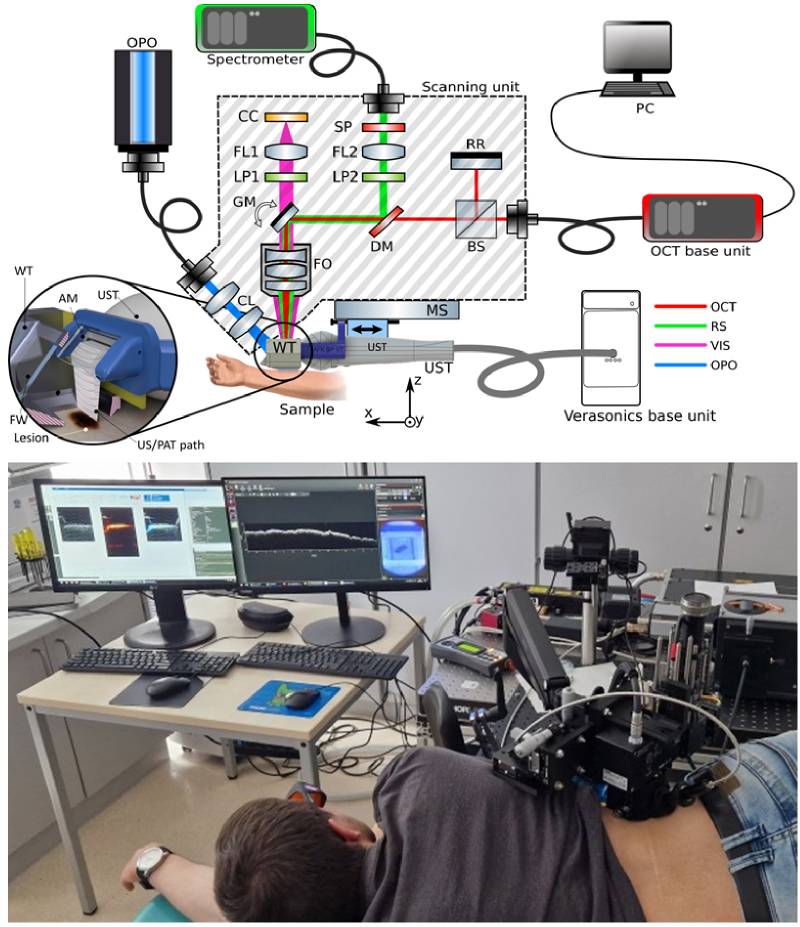
Top: Sketch of the experimental setup. Abbreviations: AM: acoustical mirror; BF: bandpass filter; BS: beam splitter, CC: camera chip, CL: collimating lens; DM: dichroic mirror; FA: fiber adapter; FL: focusing lens, FO: focusing objective; GM: (2D) galvomirrors; LP: long pass filter; MS: motorized stage; RR: retroreflextor; SP: shortpass filter, UST: US transducer; WT: water tank. The lines represent the optical paths of OCT (red), RS collection (green), VIS camera (violet), and the excitation beam for PAT/RS (blue). All the modalities measure at the same position of the WT opening. The single US/PAT B-mode imaging is performed in the y-z plane; by translating the UST together with the AM in x-direction, imaging at different planes can be performed. Bottom: application of the measurement head on a lesion at the lower back of a patient, which is placed on the examination bed with both the system and the bed being height adjustable.

The principle and functionality of parts of the multimodal device were presented earlier [33,34]. Here, we only describe the main aspects of the novel four-modal system. The experimental setup is shown in Fig. 1(top). The size of the core system is approximately 20 cm (width) x 30 cm (height) x 10 cm (depth). The system is mounted on a flexible arm so that lesions on different parts of the body of patients can be examined. US and PAT acquisition are achieved using an 18 MHz central single crystal transducer (L22-14vX-LF, Vermon, France) with an acoustical focal length of 20 mm. The transducer is connected to a research ultrasound system (Vantage 32LE, Verasonics, USA) which generates and acquires signals at a sampling rate of 62.5 MHz. The transducer is positioned horizontally within a 3D-printed resin water tank (WT), which features a square opening measuring 1 cm^2^ at the bottom. This bottom portion with the opening can be exchanged with other adapters featuring different-sized openings. For a measurement, the transducer is placed over the lesion and filled with water as an acoustic medium. The WT is equipped with a large front window (FW), through which optical excitation for PAT and RS is performed. The UST with the adapter is positioned within the WT, and the space between them is sealed with a 150 µm thick flexible membrane made from TheraBand, which allows for the motion of the UST in the x-direction. An acoustical mirror (AM), created by a glass slab positioned at a 45-degree angle reflects acoustical waves from the UST vertically onto the opening of the WT through total internal reflection, allowing for optical transparency from the top side (for optical modalities) and the front side (for PAT illumination), respectively. The efficiency of acoustic reflection was validated by comparing US intensity with and without the AM, demonstrating a highly efficient reflection rate (greater than 95%). Using threads in agar phantoms, the resolutions are measured at ≤ 200 µm (lateral), ≤ 100 µm (axial) for US; and at ≤ 300 µm (lateral) and ≤ 200 µm (axial) for PAT, which is in accordance with theoretical expectations for the system used and suitable to determine the thicknesses of many nevi [34]. As PAT and US use the same detection path, measurement at exactly the same position of the examined phantom or lesion are enabled, which also served as cross-validation for the obtained thickness values. Also, in the long-term, it can be verified whether the two modalities offer complementary information, e.g. by visualizing the vascular network around lesions which might be an additional dignity indicator.

### 2.2. Imaging and spectroscopic procedure

For 3D imaging, the transducer is translated inside the WT using a motorized stage (MTS25/M-Z8, Thorlabs, USA). The scanning head is mounted on a standard VESA monitor arm, providing support and facilitating alignment of the WT aperture with the skin surface. Optical excitation for PAT and RS is achieved with an optical parametric oscillator (OPO, SpitLight Compact 400 OPO, InnoLas Laser GmbH, Germany), generating 7 ns laser pulses at a 20 Hz repetition rate. These pulses are guided through a custom optical fiber bundle (CeramOptec GmbH, Germany) and projected via a collimator consisting of two achromatic lenses (AC127-019-A and AC254-030-A, Thorlabs, USA) and a square diffuser (ED1-S20-MD, Thorlabs, USA) onto the lesion under an angle of 50° to the surface, as indicated by the blue path in Fig. 1. Systematic tests with agar phantoms mixed with coffee, mimicking realistic melanin absorption in pigmented lesions, determined a maximum imaging depth of at least 5 mm, see also [34]. The laser power density was maintained at ≤ 6 mJ/cm^2^ per pulse, i.e. below the legal maximum permissible exposure (MPE) safety regulations [35,36].

The optical modalities (OCT and RS) are combined inside the customizable OCT probe head (OCTP-900, Thorlabs, USA), which has been modified to include a coaxial RS collection system. As the WT provides an unobstructed optical window onto the lesion, both OCT and RS can be operated directly at the same position as the acoustical modalities. The signal from a spectral domain OCT (red path) with central wavelength of 900 nm is transmitted from the base unit (GAN621, Thorlabs, USA) via fiber, divided into reference and sample arms, steered with a two-dimensional galvoscanner (GM), and focused onto the sample with a focusing objective (FO, focal length: 36 mm, OCT-LK3-BB, Thorlabs, USA). The second galvomirror, which partially transmits visible light, allows simultaneous visualization of the sample plane by projecting the opening of the WT onto a CCD camera chip (purple path in Fig. 1), filtered from the laser pulses by a short pass filter with cut off wavelength of 475 nm (Edmund Optics, United Kingdom). The OCT system's lateral and axial resolutions in air are measured with a resolution target as 4 µm and 3 µm, respectively [33].

The Raman signal (green path) is collected by the FO and decoupled from the OCT signal with a dichroic mirror (DM, DMLP805R, Thorlabs, USA) at 805 nm. Filters (LP2, 532 nm, RazorEdge ultrasteep LP Edge filter, Semrock Inc., USA, and SP, 700 nm, FESH0700, Thorlabs, USA) are used to remove the Rayleigh line and the noise from of the OCT spectrum before focusing the light onto the end facet of a fiber bundle (LLB536-IR-0,22-1, Quantum Design GmbH, Germany) and guiding it to a spectrometer (Kymera 193i, Andor Technology Ltd, UK) equipped with a CCD camera (iDus-LDC-DD, Andor Technology Ltd, UK) and a 1200 l/mm grating with 600 nm blaze. Single RS measurements were performed with 532 nm excitation wavelength, 10 s exposure time and 2.4 mJ of energy per pulse, translating to < 5 mJ/cm^2^ per pulse, compliant with MPE levels for human skin. With regard to the RS collection spot, its diameter is chosen ≤ 300 µm, as determined via full width at half maximum of the integrated signal [33]. By performing RS measurements with and without samples and, e.g., with paracetamol as reference material, it was verified that the observed Raman lines originate from the tissue samples, and not from optical components in the beam path. Possible influences of the spectral instrument function, i.e., the spectral transmission/sensitivity on the observed Rama spectra were checked and excluded.

### 2.3. Validation on skin phantoms and in clinical setting

**Fig. 2.**
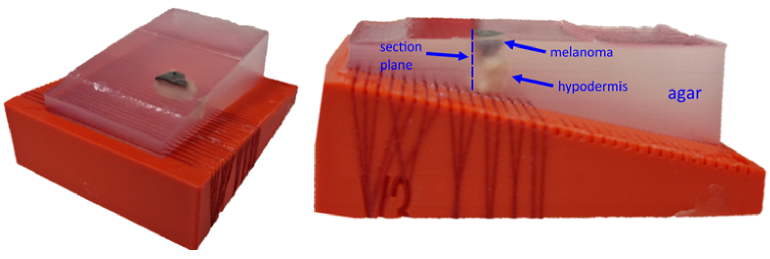
Photograph of an ex vivo melanoma sample embedded within the agar phantom. Left: the image of the phantom. Right: vertical side view, which shows the vertical view of the sample, including the different excision layers and the section plane in the middle of the excision. The red threads hold the sample in place and imitate blood vessels that are located underneath the hypodermis layer of skin.

To validate the correlation of measurements with histological data, the developed device was tested on *ex vivo* human skin samples with confirmed melanoma, obtained from the University of Rostock, under the ethics approval A 2016-0115. The samples underwent histological examination involving dehydration, fixation in formalin, and vertical sectioning at the center (from which a histology slice was taken). In addition, in order to demonstrate the influence of illumination direction on the PAT results, some of the samples were embedded in clear agar with the upper melanoma surface exposed on the top, as shown exemplarily in Fig. 2. The clear agar is transparent to optical excitation and ultrasound waves, allowing unobstructed acoustical imaging of the samples.

For demonstration of the applicability of the system in clinical environment, the setup was tested on 52 suspicious human skin lesions from 35 patients (20 males and 15 females, median age of 35 years) that were excised immediately after the *in vivo* measurements. Two neoplasms were identified as malignant melanoma cases, while the remaining 50 lesions were classified as non-malignant melanocytic nevi. All patients were of Caucasian skin type (Fitzpatrick skin types II and III). The performed measurements were approved by the Ethics Committee of the University Medical Center Rostock (A 2016-0115) and met the principles of the Declaration of Helsinki. All the participants gave their oral and written consent for participation in this study.

Patients were positioned on an examination bed for measurements. Using the camera from the OCT module, the device's opening was aligned centrally over the lesion, ensuring a waterproof seal. Alternatively, this opening can also be permanently sealed with an optically transparent polymer foil. A photograph of the device being applied to a patient is shown in Fig. 1(bottom). Despite the fact that the current version of the system was not particularly optimized for manageability in clinics, we were also able to carry out measurements on lesions located at positions with uneven surfaces such as the head or extremities. The current system requires less than 5 minutes of measurement time in total, including the application of the measurement head, the scanning during PAT and US and the integration of the Raman measurement. For each modality, between a few seconds up about 30 s are needed, depending on lesion size. In more detail, each B-mode measurement for US takes less than 0.01 s. For PAT, a B-mode measurement requires about 1 s. Depending on the number of B-modes for one lesion and the scanning step size, this amounts to 10-20 s total measurement time. The OCT measurement takes roughly 10 s, while for the Raman measurement we set an integration time of 5-10 s. For larger lesions, Raman measurements are taken at different positions with the same integration time. As the system comprises an adapter which is placed on the lesion to be measured and gently pressed against it, we did not observe motion artefacts, at the resolutions possible so far.

OCT and RS measurements were performed first, followed by filling the chamber with water for US/PAT acquisitions. Each lesion was measured with a sequence of 40 depth images (B-modes), laterally separated by 200 µm, with an acquisition time of about 30 s. These parameters were chosen to demonstrate the system functionality and are not a technical limitation. The patients reported that they did not experience any pain or discomfort during the measurements. Lesions were immediately excised post-measurement for histopathological examination, which included formaldehyde fixation, paraffin embedding, sectioning, and H&E staining. A dermatopathologist, blinded to the imaging results, provided the histological diagnosis and measured the Breslow depth for the melanoma or equivalent melanocytic infiltration depth for the non-melanoma lesions.

The US, PAT and OCT images were analyzed manually as separate grayscale stacks, with lesion thickness measured at the thickest position in each modality's stack. The largest thickness found in the images was taken as a result for each modality, in order to be compared with the Breslow depth or the infiltration depth. For US and PAT images, the OCT module's camera image was used for orientation to ensure measurements were taken at the lesion site. For reconstruction, the speed of sound was set to 1560 m/s, as measured by Weichenthal et al. [37]. Due to the limited penetration depth of the OCT system in highly pigmented nevi at 900 nm central wavelength, only thin lesions less than 500 µm thick were properly observed and measured. The contrast and dynamic range for the OCT images were automatically calculated with the OCT software, using a skin refractive index of 1.35, similar as in [11,38]. The acquisition spot for the RS measurements was manually set in the software at the center of the lesion. Reference measurements were taken adjacent to the lesion on healthy skin areas. The acquired RS spectra were processed with smoothing and normalization prior to analysis. Two versions of spectra were examined: unprocessed and filtered ones. For the latter, an iterative morphological and mollifier-based baseline correction method [39] was employed to remove the autofluorescence background.

## 3. Results and discussion

### 3.1. PAT/US imaging on *ex vivo* samples

**Fig. 3.**
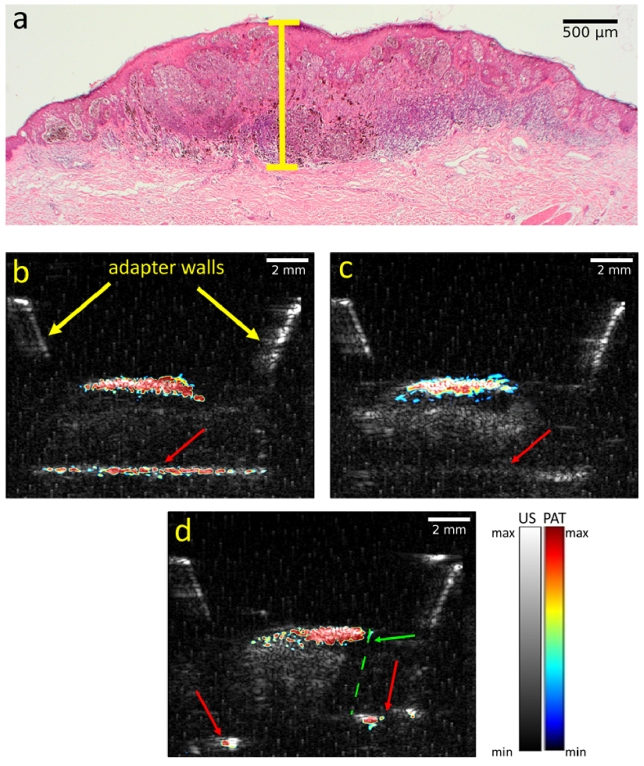
Results for the ex vivo melanoma sample. a: histological image of the melanoma sample with yellow line marking the Breslow thickness of 1.29 mm as determined by the histologist. b: combined US/PAT (532 nm excitation wavelength) measurements of the ex vivo. The US is represented in grayscale, while the PAT is represented in false color. The red arrows point at the red thread that holds the sample and mimics a blood vessel underneath the hypodermis. Measurement at the section plane with PAT illumination from the front (cut side); c: PAT illumination from the opposite direction. d: the measurement perpendicular to the section plane. The green arrow indicates the position of the edge of the sample, which has been marked with a dashed green line.

First, the dependency of PAT measurements on illumination direction using vertically cut melanoma samples was evaluated. US and PAT measurements were performed in three configurations: from the exposed front side, the back skin side (rotated 180^o^), and perpendicular to the cut edge. The aim was to analyze how the illumination direction influences the PAT measurement outcomes. The corresponding US/PAT results for one such sample are shown in Fig. 3. To ensure a consistent comparison, the dynamic range (DR) for PAT was kept constant across all illumination directions.

For the *ex vivo* melanoma sample used, PAT images displayed similar size and depth measurements from both front and back illuminations. The maximum depth of the melanocytic invasion measured was 1.25 mm, closely matching histological Breslow thickness of 1.29 mm. Front illumination resulted in an over-saturated PAT signal at the lesion, while back illumination provided a stronger signal at the bottom half of the lesion, aligning with higher melanin concentration observed in histology. Notably, the front illumination detected a red thread (imitating a blood vessel) at a depth of approximately 4.5 mm, whereas back illumination did not penetrate deeply enough to visualize the thread, which was only resolved with US. The perpendicular PAT measurements are comparable with the front and back illumination results in terms of depth and provided additional information on lesion width. The thread signal appeared at the sides of the sample but was not as prominent as in the front illumination. These results suggest that while PAT is effective in measuring lesion depth including melanoma and detecting subdermal features, the illumination angle significantly impacts the quality and depth of the imaging results. For the measurements in this work, the illumination angle was always at 50° to the surface.

### 3.2. PAT/US imaging *in vivo*

**Fig. 4.**
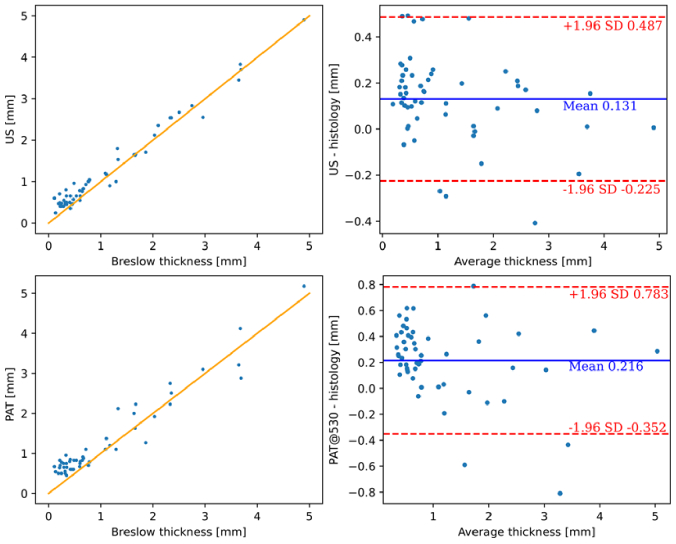
Clinical results for US (top) and PAT with excitation wavelength of 532 nm (bottom). The plots on the left illustrate the (in vivo measurement vs. equivalent histological thickness), with yellow lines indicating the 1:1 agreement line; the plots on the right show the corresponding Bland-Altman plots. The blue line represents the mean difference between the two modalities, while the dashed red line illustrates the standard deviation (SD).

**Fig. 5.**
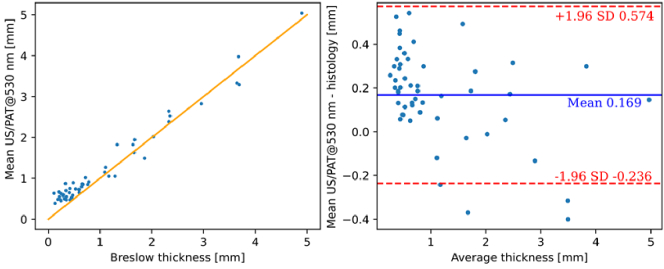
Clinical results for averaged US/PAT (532 nm excitation wavelength), which yield the best results in terms of correlation to the histological thickness. Left: plot depicting the in vivo measurement result vs. equivalent histological thickness, with yellow lines indicating 1:1 agreement line. Right: the corresponding Bland-Altman plot. For the left plot, the correlation coefficient R^2^ is calculated at 0.97.

To show that the system can be used in clinical settings, *in vivo* lesion thickness measurements were performed and the resulting data and corresponding Breslow or infiltration thicknesses are presented in Fig. 4 using scatter plots and Bland-Altman plots, commonly utilized to compare clinical measurements from different modalities [40]. The US modality exhibits the narrowest confidence interval (CI) of approximately 0.7 mm. In contrast, PAT at 532 nm shows a larger deviation from histological values, with a CI of 1.1 mm. While US alone shows the highest correlation and CI as a single modality and with the current setup, the averaging of the thickness results from US and 532 nm PAT provides the most accurate prediction of infiltration depth or histological Breslow thickness, respectively, yielding a mean deviation of +0.17 mm and a CI range of 0.8 mm, as illustrated in Fig. 5. The combined results deliver a promising coefficient of determination (R^2^) of 0.97.

On average, both modalities tend to overestimate the thickness compared to the Breslow depth by 0.1 to 0.2 mm. This trend is consistent with other clinical studies using non-invasive methods [11,20–22,41]. Those studies also report similar overestimations, however for lesions thinner than 0.5 mm, and slight underestimations for thicker lesions. The observed overestimation for thin lesions can be attributed to tissue shrinkage, which occurs immediately after excision and is exacerbated during the dehydration and paraffinization stages, leading to a reduction in histological thickness [28,40–44]. Additionally, histology might underestimate overall lesion thickness since the excision slice is not necessarily taken at the lesion's greatest depth, whereas our modalities provide a comprehensive set of B-mode images for analysis. Notably, at the critical thickness of around 1 mm, which is the cutoff depth for performing a sentinel node biopsy in case a melanoma is diagnosed, our system exhibits superior performance compared to other modalities. The slight underestimation for thicker lesions could be due to variations in the speed of sound between individual tumors and different sites of normal skin [37], which affects the accuracy of both US and PAT predictions.

The *in vivo* capability indicates that our system may be a valuable tool to assist dermatologists in measuring lesion size and determining excision margins. The spatial resolutions of our system are higher than those of other similar US/PAT devices reported in the literature, which are primarily based on commercial US systems and have been used in various applications but not extensively applied to skin cancer so far [45–47].

### 3.3. OCT imaging *in vivo*

**Fig. 6.**
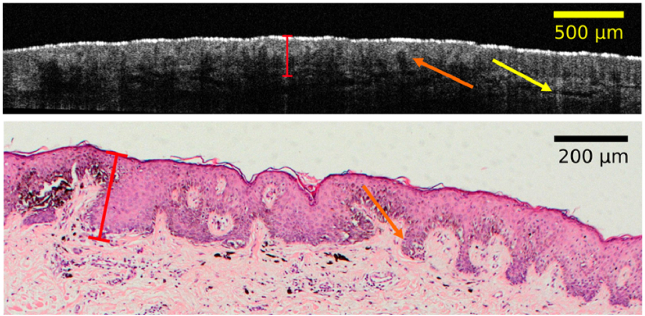
Example OCT measurement on a lesion. Top: in vivo 2D OCT measurement across the center of the lesion, with manual measurement of the deepest invasion of 270 µm. The orange arrow points at the particular rete ridges structures, which are also visible in the corresponding histological evaluation. The yellow arrow points at the dermal epidermal junction. Bottom: the corresponding histological evaluation, with marked equivalent depth of 247 µm.

**Fig. 7.**
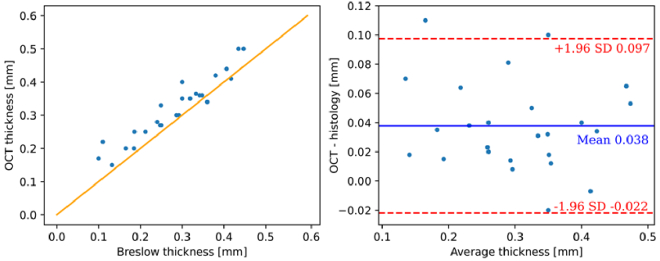
Clinical results for OCT measurements. The plot on the left illustrates the in vivo measurement vs. equivalent histological thickness, with yellow lines indicating the 1:1 agreement line; the plot on the right shows the corresponding Bland-Altman plot.

Similarly, the OCT measurements were manually evaluated by identifying the largest lesion depth, as shown in Fig. 6. The contrast/DR for OCT images was calculated automatically with the OCT software, and the refractive index for skin was set to 1.35, as in similar reports [11,38]. As illustrated, the OCT is capable of resolving the depth of invasion of thin lesions and also of capturing some morphological details from the epidermal layer, including the rete ridges and the dermal-epidermal junction. These features could potentially be used in the future for aiding the diagnosis of melanoma. In particular, it should be noted, that certain variants of OCT (e.g., LC-OCT) can enable cellular resolution, and, thus, represent an interesting extension for our system in the future. The thickness results of OCT *in vivo* measurements are presented in Fig. 7. In contrast to the acoustic modalities PAT and US, the utilized OCT is limited in terms of penetration depth, with the thickest lesion which can reliably be observed exhibiting a thickness of around 0.5 mm. In our previous study [11,48], we used an OCT system at 1300 nm and observed similar limitations at slightly larger depths of around 650 µm and slightly lower resolution. For example, the above rete ridges could not be observed with that system. Similar to the findings observed with PAT and US, OCT tends to overestimate the thickness in comparison to histology (probably again due to tissue shrinkage during preparation of the histological examination), with an average discrepancy of approximately 40 µm. Conversely, OCT exhibits a considerably improved agreement with histology, with a confidence interval of approximately 120 µm.

While OCT showed the best correlation to histological thickness, the limitation in penetration depth is due to high optical absorption and scattering in highly pigmented nevi. Although initially intended for non-invasive classification and diagnosis of skin lesions by evaluating morphological features (similar to histological images), due to the small number of measured melanomas in this study, the current OCT images provided limited diagnostic information. With a larger dataset comprising more malignant samples, it would be possible to train deep neural networks, such as convolutional neural networks (CNNs), which have shown promising results in skin cancer classification [49] and image segmentation [50,51].

In comparison to other studies that utilized US imaging [11,41] and OCT [11], which primarily analyzed thin lesions (≤ 1 mm) due to the limited penetration depth of OCT or HFUS, this study encompasses a significantly broader range of lesion thicknesses (0.1-5 mm). Conversely, other studies that investigated thicker lesions *in vivo* using single B-mode images reported CI values exceeding 2 mm [19,21,52], which is considerably larger than the CIs achieved by our presented system.

### 3.4. Raman spectroscopy *in vivo*

**Fig. 8.**
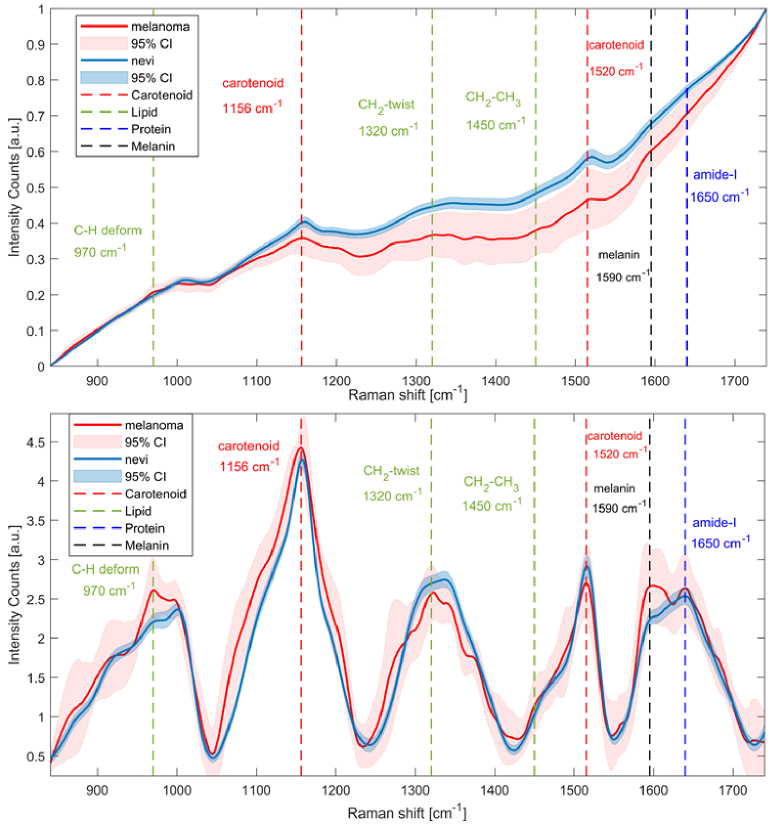
Example Raman spectra of melanoma and non-cancerous skin nevi with the 95% confidence interval in the fingerprint range of 800 - 1800cm^-1^. The vertical dashed lines mark the wavenumbers corresponding to specific characteristic chemical signatures in the fingerprint region that are commonly used as markers for dignity establishment. Top: original spectra including the autofluorescence background, bottom: with baseline correction.

Finally, example results for RS measurement are presented in Fig. 8. By keeping the autofluorescence, the peaks for carotenoids, proteins, lipids, and melanin in the skin are discernible but only marginally above the background level. In particular, carotenoid lines could also be observed, as the Raman excitation wavelength was chosen at 532 nm, close to the carotenoid (pre-)resonance; these lines are usually not observed when the excitation wavelength is chosen in the infrared range, as is usually the case for typical Raman setups. Notably, the fluorescence intensity of the melanoma cases investigated here is similar to nevi at the lower end of the fingerprint region (800 - 1100 cm^-1^) but is significantly lower at the central fingerprint region (1150 - 1650 cm^-1^). We found that this trend is consistent when measuring at different positions of the same lesion, even though the number of melanoma available for our measurements in this phase was low.

With the fluorescence background removed, the spectra of melanoma revealed significantly higher intensities at 1590 cm^-1^ (melanin) and 970 cm^-1^ (lipids, C_2_ H deformation) compared to non-cancerous melanocytic nevi. The intensity of the amide-I band (proteins) at 1650 cm^-1^ did not show significant variation across the different skin spectra. The latter results are consistent with similar RS measurements on skin lesions [23,53]. Overall, it appears to be possible that in future, apart from the known skin lines including the carotenoid lines (not accessible at infrared Raman excitation wavelengths), the autofluorescence levels may be used as parameter for skin lesion assessment and diagnostics.

### 3.5. Discussion of system performance

While the initial findings are promising, more measurements on melanoma and other types of skin cancer samples are required to conduct a proper statistical analysis. The current study was limited by a small melanoma samples, which hinders comprehensive statistical validation with regard to diagnostic accuracy. Future studies will focus on increasing the number of melanoma samples to enable a robust analysis and improve the reliability of the RS findings. Moreover, combining the predictions from RS data with information from the OCT modality as based on morphological details, could enhance the classification and diagnosis of melanoma (or other types of skin cancer). By combining the spectral data from RS with the structural imaging provided by OCT, and utilizing advanced machine learning techniques like CNNs, potentially reliable discrimination between melanoma and non-melanoma lesions can be achieved compared to using data from RS alone. CNNs have already shown promising results in both classification [49] and image segmentation tasks for skin cancer images [50,51]. It can be expected that a multimodal approach could further improve diagnostic accuracy. In particular, it will be interesting to analyze the combined diagnostic accuracy for all four modalities or combinations thereof under the framework of a comprehensive machine learning approach which was not part of this work. Furthermore, it is intended to perform PAT measurements with additional excitation wavelengths. By utilizing longer wavelengths, it is possible to enhance the penetration depth of the excitation light, although this may result in a reduction in PAT signal intensity due to increased absorption. For our measurements so far, we found that the wavelength region at 532 nm was optimal considering the optical components involved, the fibre bundles available, the Raman spectrometer bandwidth, and the beam paths to overlap with the other modalities. In future, the PAT signals generated by varying the laser wavelength can be employed as a means of photoacoustic spectroscopy, which provides further insights into the lesion [54].

Currently, the resolution of the ultrasound and photoacoustic units is 100 and 200 µm in axial direction, respectively, limited by the ultrasound transducer available for this work. For melanomas, the critical tumor thickness for metastasis is 1 mm. Typically, histological thickness determination has an accuracy of below 100 µm. Increase in resolution for our setup can be achieved by using ultrasound transducers with more transducer elements and smaller step size (in this work, 200 µm) for the scanning procedure during measurement. If the step size is considerable smaller than the resolution of the PAT and US modalities, more advanced reconstruction approaches can be utilized to improve the imaging and eventually extract additional morphological features from the measurements. The OCT system has a higher resolution of 3 µm in axial direction, whereas the penetration depth is only at 500 µm. By using, for example, line-field OCT systems even cellular resolution can be achieved [13,14], so that it appears feasible to extract more details morphological and diagnostic parameters from the OCT measurements, potentially increasing lesion classification. For the Raman modality, a laser spot diameter of <300 µm is currently used, to be able to acquire the integrated signal from smaller lesions followed by the signal from adjacent normal skin. In a further step, the size of the laser spot will be optimized to achieve a better sampling across the lesion area and still remain below the MPE levels required for in vivo measurement. We expect that these perspectives will improve the capabilities of our system so that differential diagnosis for different types of skin cancer including melanoma can be achieved. As malignant melanomas have considerably more heterogeneous surfaces and cell distributions than nevi and their tumor lower limit is much more difficult to determine because melanophages and inflammatory infiltrates have to be distinguished from the actual tumor cells, we will evaluate the capabilities of our system also with respect to these aspects, once more cases of melanoma and other types of skin cancer are investigated in the next phase.

Our measurement system, which integrates US, PAT, OCT, RS, and camera modalities in a novel manner, shows a high correlation with histopathological standards and has promising capabilities in clinical settings. Nevertheless, further development of the device would be advantageous. Potential future enhancements include application of USTs with higher central frequency and/or more transducer elements to improve the resolution of the acoustic capabilities, implementation of PAT illumination from additional angles to improve imaging accuracy, integration of artificial intelligence for automatic segmentation, analysis and classification of lesion. Also, the system will profit from automated co-registration of the different imaging modalities, representing an important step towards fast and ideally close to real-time analysis of the data. These advancements could ultimately enable high diagnostic accuracy and usability of the system, ultimately providing the dermatologists an effective, more objective and non-invasive skin cancer diagnosis tool.

## 4. Conclusions

In this work, we developed a four-modal system that integrates US, PAT, RS and OCT in a single measuring head for skin tissue evaluation with the aim to show its capability for measuring the infiltration depth and dignity of lesions and the long-term goal to detect malignant lesions such as melanoma in vivo. The performance of the developed multimodal imaging system was evaluated using *ex vivo* melanoma samples embedded in agar blocks, demonstrating its capability in resolving melanocytic invasion thickness. Following that, in *in vivo* measurements at the clinical setting, skin lesions were examined at below the legally permitted MPE levels for the employed electromagnetic radiation, with acquisition times under five minutes per nevus for all measurements together. It is important to note that the measurements with the individual modalities typically required just a few up to a few ten seconds at this stage. Immediate excision and histopathological analysis showed good correlation between the imaging modalities (US, PAT with 532 nm excitation, and OCT) and histological Breslow or infiltration thickness, for thin lesions (≤ 5 mm). Despite slight overestimation due to tissue shrinkage, the combined US and PAT at 532 nm achieved a promising R^2^ of 0.97. RS measurements indicate that, even with autofluorescence, peaks for carotenoids, proteins, lipids, and melanin are discernible above the background level. Removing the fluorescence background shows higher intensities for melanoma at melanin and lipid lines as compared to non-cancerous nevi. This opens the possibility to use the ratio between characteristic Raman lines of the skin, e.g. proteins and lipids, together with the autofluorescence as diagnostic criteria, once a larger data set is available.

The multimodal system integrating US, PAT, OCT, RS, and camera modalities shows that it may provide the required information with respect to infiltration depth and dignity to be used for skin cancer diagnosis. It allows for fast, non-invasive measurements, and might be helpful for the surgeon with the decision on excision margins. We believe that the combination of the modalities is necessary for multiple reasons: in general, OCT can deliver infiltration depth information until a certain lesion thickness, but potentially more morphologic features when modern OCT systems are used. US and PAT measure infiltration depth, but cannot deliver diagnostically relevant information at this stage, which is only possible with Raman spectroscopy, with molecular specificity. All the modalities may strongly benefit from new technological developments or improved algorithms for analysis in future. For this work, our main intention was to show the basic operability and functionality of the developed system in the clinic. We could not cover all different skin types at this stage. This, however, may be important as the system performance might differ between the different skin types. Therefore, this evaluation will be part of our future work. Also, it might be the case that at much higher resolution, which is possible when using modern transducer technology, motion artefacts (e.g. blurring) will be observed and need to be addressed. So far this was not a limitation, however. Thus, routine measurements on patients appear feasible. Additionally, manageability can further be increased, in particular when in a future iteration step the measurement head will be miniaturized by using micro-optical elements and smaller mechanical holders which was not in the focus of this work.

Future technological enhancements could include application of USTs with higher frequencies, additional PAT illumination angles, AI integration for automatic lesion analysis and segmentation, system miniaturization and further in vivo measurements on lesions, all aimed at enabling a high diagnostic accuracy and clinical usability. Despite the fact that the accuracy of the individual modalities can still be improved, it can be expected that the evaluation of all four modalities using algorithms from artificial intelligence might enhance the classification accuracy demonstrating the diagnostic value of the system. The presented results thus show that a multimodal approach can be useful towards establishing a non-invasive optical biopsy for in vivo skin diagnostics.

## Data Availability

Data underlying the results presented in this paper are not publicly available at this time but may be obtained from the authors upon reasonable request.
